# Characterization of Ex Vivo and In Vitro Wnt Transcriptome Induced by Spinal Cord Injury in Rat Microglial Cells

**DOI:** 10.3390/brainsci12060708

**Published:** 2022-05-30

**Authors:** Carlos González-Fernández, Pau González, Francisco González-Pérez, Francisco Javier Rodríguez

**Affiliations:** Laboratory of Molecular Neurology, Hospital Nacional de Parapléjicos, 45004 Toledo, Spain; paug@sescam.jccm.es (P.G.); francisco.gzlez@gmail.com (F.G.-P.); fjrodriguez@sescam.jccm.es (F.J.R.)

**Keywords:** Wnt, frizzled, microglia, macrophage, spinal cord injury, neuroinflammation

## Abstract

It is well known that inflammation is crucial in the onset and progression of neurodegenerative diseases and traumatic central nervous system (CNS) injuries, and that microglia and monocyte-derived macrophages (MDMs) play a pivotal role in neuroinflammation. Therefore, the exploration of molecular signaling pathways that are involved in the microglia/macrophage response might help us to shed light on their eventual therapeutic modulation. Interestingly, there is growing evidence showing that the Wnt family of proteins is involved in different neuropathologies that are characterized by a dysregulated neuroinflammatory response, including spinal cord injury (SCI). Here, we aimed to validate a methodology with competence to assess the physiologically relevant Wnt expression patterns of active microglia and MDMs in a rat model of SCI. For that purpose, we have selected and adapted an in vitro system of primary microglia culture that were stimulated with a lesioned spinal cord extract (SCE), together with an ex vivo protocol of flow cytometry sorting of rat microglia/MDMs at different time-points after contusive SCI. Our study demonstrates that the expression profile of Wnt-related genes in microglia/MDM cells exhibit important differences between these particular scenarios which would be in line with previous studies where similar discrepancies have been described for other molecules. Moreover, our results provide for a first experimental report of the Wnt transcriptome in rat microglia and MDMs after SCI which, together with the research platform that was used in the study, and considering its limitations, we expect might contribute to foster the research on Wnt-driven immunomodulatory therapies.

## 1. Introduction

Microglia are the resident immune cells of the central nervous system (CNS) that are responsible for the surveillance and defense against pathogens and CNS disorders, but they also play critical homeostatic functions under physiological conditions, including the control of neuronal excitability, synaptic organization, trophic support, and debris removal by phagocytosis [1,2,3,4,5]. When an injury or an inflammatory stimulus take place in the CNS, microglia is the earliest cell type to respond. They lose their ramified phenotype, adopting a rounded/amoeboid morphology strongly resembling monocyte-derived macrophages (MDMs), and release both pro- and anti-inflammatory mediators such as reactive oxygen species, cytokines, and chemokines [1,4,5,6].

It is well known that inflammation plays a critical role in the onset and progression of neurodegenerative diseases and traumatic CNS injuries [7,8], including spinal cord injury (SCI) [1,9], which is a devastating neuropathological condition that leads to a severe and irreversible loss of function and permanent disability, with substantial socioeconomic repercussions, and for which there are still no effective treatments [10,11]. The SCI pathophysiology has been commonly described as the result of two sequential and mechanistic distinct stages: the initial mechanical damage to the spinal cord, leading to the death of neurons and glial cells and known as primary injury; and the so-called secondary injury, which begins within minutes following the initial insult and continues for weeks or months. This secondary phase involves a series of cellular, molecular, and biochemical phenomena that ends in a progressive self-destruction of the tissue larger that is than the primary injury, which hampers concurrent endogenous neuroprotective and regenerative mechanisms and thus functional recovery [10,11,12]. Among them, microglia plays a master role by its rapid but mostly pro-inflammatory steady activation after injury, with the release of diverse chemokines and cytokines that contribute to secondary damage and promote the recruitment of peripheral circulating myeloid cells such as neutrophils and monocytes. However, both microglia and MDMs also confer beneficial effects through the acquirement of a transient and less pronounced M2 phenotype that is involved in the anti-inflammatory factors release, the clearance of debris, and the attempt to contain the lesion size during the acute stages of the injury. Overall, a final lack of success resolving inflammation leads to a long-lasting pro-inflammatory response with aberrant tissue remodeling, and subsequently the neurological dysfunction worsens than what would be expected from the primary insult that characterizes SCI [1,4,5,6,13,14,15,16].

In this context, the exploration of those molecules that are implicated in neuroinflammation, particularly in the SCI-associated microglia/macrophage response modulation, might help us to shed light on the pathological mechanisms underlying the progress and outcome of this neuropathological condition. Interestingly, over the last years there is growing evidence showing that the Wnt family of proteins play a relevant role in different neuropathologies that are characterized by a dysregulated neuroinflammatory response [17,18,19,20], including SCI [21,22,23,24,25,26,27,28,29,30,31]. However, very few studies have evaluated the expression of the Wnt family of proteins in microglial cells. More specifically, it has been shown that cultured microglia are able to express different Wnt receptors and co-receptors [32,33], in accordance with studies demonstrating that this cell type is able to respond to Wnt signals [33,34,35,36,37,38,39,40,41,42,43,44]. Remarkably, there is some evidence pointing to a divergent modulation of the microglia/MDM cells inflammatory response to Wnt signals depending on the cellular or physiological context. Some authors have described a role for canonical Wnt/β-catenin signaling ligands such as Wnt1 and Wnt3a in the blockage of inflammatory microglial activation and/or proliferation in vitro [45,46] and in vivo [47], while other studies reported a stimulatory effect in proliferation [48] and also in survival of these cells in vitro [48,49,50]. However, there are also in vivo studies that have shown a lack of significant changes in microglial reactivity when a Wnt3a treatment is applied on CNS damage animal models [51,52]. Added to this already complex scenario, there are also conflicting evidences regarding independent Wnt/β-catenin signaling, and non-canonical ligands such as Wnt5a are able to induce proliferation and/or activation of microglial cell lines [32,53], while sobreexpressed Wnt5a in the spinal cord of rats after SCI did not induce significant changes in microglial reactivity [54].

In this line, it should be noted that most of the current experimental evidences on the Wnt roles on the microglia cell response have been described in cultured mice microglial cells that are stimulated with bacterial lipopolysaccharide (LPS) or a single cytokine such as Interleukins (IL), Tumor Necrosis Factor Alpha (TNFα), or Interferon-γ (IFNγ) [4,5,55,56,57]. Nevertheless, after SCI microglial activation is triggered as a result of a wide and dynamic post-injury cocktail of cytokines and damage-associated molecules [4,5,58]. In this regard, in another recent publication we described whether the use of a 24 h post-SCI protein extract (SCE) to stimulate rat astrocytes in cell culture induced alterations on their Wnt transcriptome [59]. Moreover, this methodology has been further proven to activate rat microglia cell cultures in a more physiological way than LPS or a concrete pro-inflammatory cytokine, based on its higher correlation for specific markers of activation that were observed in microglia and MDM cells that were analyzed by flow cytometry [60]. On the other hand, it also should be noted that significant between rodent and human species-related differences on the SCI pathophysiology and by extension microglial cells has also been described [4,5,57,61]. Indeed, in previous studies we analyzed the Wnt transcriptome of the healthy and injured spinal cord of mouse and rat, as well as uninjured human spinal cord [26,27,28,29,30,31,62]. As expected, we found some remarkable differences in the response to SCI between mice and rats at both the whole transcriptome and the immunohistochemical characterization of the cell-type expression pattern of few Wnt receptors and co-receptors. Nonetheless, most of our studies have been performed in rats, since its higher resemblance to human pathophysiology after contusion SCI [11,63].

Based on the aforementioned observations, in the present study we examined the differential gene expression pattern of the main Wnt family members (including ligands, receptors, and soluble modulators) by adapting two independent experimental approaches: (i) an in vitro system of primary microglia culture that was stimulated with LPS or SCE from 24 h post-injury tissue (hpi), and (ii) an ex vivo protocol of flow cytometry sorting allowing isolation of microglia/MDMs cells from rat spinal cord at different time-points after contusive SCI and subsequent mRNA extraction. By using those methods, we examined whether these ex vivo and in vitro approaches might contribute to determine physiologically relevant differences in the patterns of expression of the main Wnt family members between rat non-activated (NA) and activated microglial cells, and pave the way for a reliable and versatile research platform to foster the assessment of potential immunomodulatory Wnt therapies.

## 2. Materials and Methods

### 2.1. Animals and Surgical Procedures

A total of 27 adult male (3 months; weight approximately 300 g) and 66 adult females (3 months; weight approximately 250 g) Wistar rats were used to obtain the SCE for in vitro assays and spinal cord microglial cells for ex vivo experiments, respectively. The animals were obtained from our in-house colony, and grown and maintained in our animal facilities. Animal housing and experimental procedures were conducted following the Spanish (Royal Decree 53/2013) and the European Union (2010/63/EU) directives and were approved by the Bioethics Committee at The National Hospital of Paraplegics (Toledo, Spain) (Permit numbers 51/2009 and 45/2008). The contusive spinal cord lesions and post-operative cares were performed as we have described in previous reports [26,27,28]. All efforts were done during the whole experimental process to minimize animal suffering.

### 2.2. Primary Microglia Cell Cultures and Stimulation with SCE

#### 2.2.1. Preparation of the SCE

In order to simulate the damage-associated molecular pattern of the microenvironment occurring at the acutely injured spinal cord, we obtained the SCE at 24 hpi, when numerous harmful stimuli signals have been described to be increased after SCI [64], following the previously described protocol [59]. Briefly, the lesioned animals were intra-aortically perfused with 150 mL of sterile heparinized saline solution and straight away a 1 cm of spinal cord containing the lesion epicenter was dissected, homogenized in Dulbecco’s Modified Eagle Medium (DMEM) (41965039, Thermo Fisher Scientific, Madrid, Spain) (1 mL/cm of lesioned spinal cord) using an Ultra-Turrax homogenizer (8002001, IKA, Staufen, Germany), sonicated (5 sonication pulses of 20 s interspersed by 30 s stops) with an ultrasonic homogenizer (UP50H, Hielscher, Teltow, Germany), and centrifuged at 12,000× *g* for 5 min at 4 °C. The supernatant containing the soluble proteins was recovered and stored at −20 °C until use. Quantification of the protein content was performed by using the Bradford Protein Assay (23236, Thermo Fisher Scientific, Madrid, Spain).

#### 2.2.2. Microglial Cell Cultures

The primary microglia cells cultures were prepared from the cerebral cortex of 75 neonatal Wistar rats at P0–P2. Briefly, the pups were decapitated and the cerebral cortices were immediately dissected out, the meninges and blood vessels were carefully removed, and the tissue was incubated during 20 min at 37 °C in 0.25% trypsin (25200056, Thermo Fisher Scientific, Madrid, Spain). The cortices were then mechanically triturated using a glass pipette and filtered through a 70 μm nylon mesh (11597522, Fisher Scientific, Madrid, Spain) in the presence of 100 U/mL of DNAse (11284932001, Sigma-Aldrich, Steinheim, Germany). Subsequently, cells were collected by centrifugation at 200× *g* during 10 min, resuspended in DMEM (41965039, Thermo Fisher Scientific, Madrid, Spain); supplemented with 10% of fetal bovine serum (FBS) (10270, Thermo Fisher Scientific), 7.5 mM of HEPES buffer (15630056, Thermo Fisher Scientific, Madrid, Spain), 100 U/mL of penicillin and 100 μg/mL of streptomycin (15070063, Thermo Fisher Scientific, Madrid, Spain); and plated at a density of 6 × 10^5^ cells/cm^2^ in 175 cm^2^ culture plastic flasks (178883, Thermo Fisher Scientific, Madrid, Spain) that were previously coated with poli-D-lysine (20 μg/mL) (P7280, Sigma-Aldrich, Steinheim, Germany). The cell cultures were then incubated at 37 °C in a humidified atmosphere of 5% CO_2_ and 95% air. The medium was replaced at 3 days in vitro (DIV) and thereafter every 7 DIV. At 3–5 days after the cultures became confluent (13–15 DIV), the microglia cells were separated by shaking the flasks at 250 rpm at 37 °C for 3 h and collected by centrifugation at 200× *g* during 10 min. Immediately after, the cells were incubated during 30 min at 4 °C with a monoclonal mouse anti-rat CD11b phycoerythrin-conjugated antibody (562105, BD Biosciences, San Jose, CA, USA) that was diluted to 1:250 in phosphate buffered saline (PBS) (10010015, Thermo Fisher Scientific, Madrid, Spain) containing 0.5% of bovine serum albumin (BSA) (A7906, Sigma-Aldrich, Steinheim, Germany), 2 mM of EDTA (E5134, Sigma-Aldrich, Steinheim, Germany), 15 mM of HEPES buffer (15630056, Thermo Fisher Scientific, Madrid, Spain), and 1% FBS (10270, Thermo Fisher Scientific, Madrid, Spain). Subsequently, the cells were collected by centrifugation at 380× *g* during 2 min and resuspended in Hank’s Balanced Salt Solution without calcium and magnesium (HBSS -/-) (14175053, Thermo Fisher Scientific, Madrid, Spain) containing 0.5% of BSA (A7906, Sigma-Aldrich), 2 mM of EDTA (E5134, Sigma-Aldrich, Steinheim, Germany), 15 mM of HEPES buffer (15630056, Thermo Fisher Scientific, Madrid, Spain), and 1% FBS (10270, Thermo Fisher Scientific, Madrid, Spain). CD11b-positive cells were purified by flow cytometry in a FACS Aria Cell Sorter (BD Biosciences, San Diego, CA) and recovered in FBS that was supplemented with 15 mM of HEPES buffer (15630056, Thermo Fisher Scientific, Madrid, Spain) and 100 U/mL of penicillin and 100 μg/mL of streptomycin (15070063, Thermo Fisher Scientific, Madrid, Spain). Finally, microglial cells were plated into 24-well culture plates (142475, Thermo Fisher Scientific, Madrid, Spain) at a density of 60,000 cells/well for purity and morphology evaluation, or into 12-well culture plates (150628, Thermo Fisher Scientific, Madrid, Spain) at a density of 120000 cells/well for quantitative Real-Time PCR (qRT-PCR) assays, and maintained during 24 h before ready for further treatment. The activation of microglial cells was performed by exposing the cell cultures to 200 µg/mL of SCE or 100 ng/mL of LPS as reference of pro-inflammatory microglia activation (L1887, Sigma-Aldrich, Steinheim, Germany), which were already shown in a previous study as competent to activate primary cultures of rat astrocytes [59]. Following the same procedure that was used with astrocytes, the medium was replaced at 24 h post-activation (hpa) to completely remove either LPS or SCE. Next, qRT-PCR-based analysis was used to investigate the gene expression changes of Wnt-related molecules at 24 hpa and 3 days post-activation (dpa).

#### 2.2.3. Assessment of Microglia Cell Culture Purity

Microglia cell purity was performed by ionized calcium binding adaptor molecule 1 (Iba1) immunocytochemistry, as previously described [59]. We used a polyclonal rabbit anti-Iba1 primary antibody (019-19741, Wako, Osaka, Japan) (1:500) and the corresponding Dylight 488-linked goat anti-rabbit secondary antibody (Ab96899, Abcam, Cambridge, UK) (1:500). The cells that were processed without the primary antibody were used as controls for immunocytochemistry and no non-specific staining was observed. Following, 40 20× magnification images per well were obtained in an In Cell Analyzer 1000 Imaging and Analysis System (1347366, GE Healthcare). We quantified the number of DAPI^+^ nuclei/Iba1^+^ cells by using the analysis tools of the In Cell Analyzer 1000 Imaging and Analysis System (Figure S1). From these data, the culture purity was determined as a percentage of DAPI^+^/Iba1^+^ cells with respect to the total DAPI^+^ nuclei in the range of 92–98%. This analysis was performed in triplicate in each culture and 3 independent cultures were used.

### 2.3. Microglia Isolation from Spinal Cord Tissue

The isolation of microglial cells from both non-lesioned (NL) and lesioned adult rat spinal cord at 24 hpi and at 3, 7, and 14 days post-injury (dpi) was carried out as described in previous reports with slight modifications [60,65]. Briefly, the anesthetized animals were intra-aortically perfused with 150 mL of heparinized saline solution. A 3-cm-long thoracic spinal cord fragment from NL or 1-cm-long spinal cord fragment containing the wound epicenter from the injured animals was dissected and, before initiating the dissociation process, 3 different spinal cord fragments per time-point that was evaluated were pooled to generate each final sample. Finally, five independent pools were used for each evaluated time-point. From here, all the procedures were carried out on ice. After removal of the meninges in Hibernate Medium (A1247501, Thermo Fisher Scientific, Madrid, Spain), the spinal cord segments were mechanically dissociated with fine scissors in 3 mL Hibernate supplemented with 2% B27 (17504044, Thermo Fisher Scientific, Madrid, Spain) and 0.8% Glutamax (35050038, Thermo Fisher Scientific, Madrid, Spain), and the tissue was then incubated in the presence of 2.5 mg/mL of collagenase D (11088858001, Sigma-Aldrich, Steinheim, Germany) and 100 U/mL of DNAse (11284932001, Sigma-Aldrich, Steinheim, Germany) for 40 min at 37 °C. Immediately after, an additional mechanical trituration step with a 1 mL pipette was done before stopping the enzymatic digestion by adding 8 mL of DMEM (41965039, Thermo Fisher Scientific, Madrid, Spain) plus 10% FBS (10270, Thermo Fisher Scientific, Madrid, Spain). The suspension was then filtered through a 70 μm cell strainer, pelleted at 170× *g* for 5 min at 20 °C, and the supernatant was carefully aspirated. Subsequently, the cell pellet was resuspended in 70% Percoll (P4937, Sigma-Aldrich, Steinheim, Germany) at room temperature and transferred to a new tube. Slowly, 5 mL of Percoll 37% were layered on top of the cell suspension, and then centrifuged (with no brake) at 500× *g* for 20 min at 20 °C. Afterwards, the top layer containing the myelin debris was carefully removed and the interphase was collected in a new tube, with 10 mL of fresh HBSS -/- buffer (14175053, Thermo Fisher Scientific, Madrid, Spain). After centrifugation at 670× *g* for 2 min at 4 °C, the supernatant was discarded and the resulting cell pellet was resuspended in 300 μL of HBSS -/- buffer (14175053, Thermo Fisher Scientific, Madrid, Spain) plus 10% FBS (10270, Thermo Fisher Scientific, Madrid, Spain) for the quantification in a hemocytometer.

The antibody labelling protocol was carried out in two steps. First, the cell suspension was incubated 5 min at 4 °C with an unconjugated mouse anti-rat CD32 antibody (550270, BD Biosciences, San Jose, CA, USA) (1:50) for Fc-blocking. Then, a combination of antibodies against well-established mononuclear cell markers was added and incubated for 30 min at 4 °C: (1) mouse anti-rat CD11b PE-conjugated (562105, BD Biosciences, San Jose, CA, USA) (1:250) for purification of the microglia/macrophage cells as the predominant mononuclear CD11b^+^ population at all times assessed; (2) mouse anti-rat CD45 FITC-conjugated (11-0461-80, Thermo Fisher Scientific, Madrid, Spain) (1:250) for differentiation of microglia (low CD45^+^) and macrophage (high CD45^+^) cell subpopulations; and (3) rabbit anti-rat PolyMorpho Nuclear Neutrophil (PMN) FITC-conjugated (AIFAD51140, Accurate Chemical & Scientific Corporation, Westbury, NY, USA) (1:100) for elimination of neutrophils, the second largest mononuclear CD11b^+^ cell population. Following, the samples were centrifuged at 2000 rpm for 5 min at 4 °C, the supernatant was discarded and the cells were resuspended in 300 μL of HBSS -/- (14175053, Thermo Fisher Scientific, Madrid, Spain). Optimization of the protocol to obtain a high cell yield and purity of microglia/macrophage cell populations was performed in a FACSCantoII^TM^ (BD Biosciences, San Diego, CA) by excluding cell fragments and debris (SSC-A vs. FSC-A), identifying singlet events (FSC-W vs. FSC-H), and gating on CD11b, CD45, and PMN immunolabelled populations. The optimized protocol that was used for mRNA extraction excluded CD45 sorting, since we were only able to discriminate low and a high CD45^+^ subpopulations over the CD11b^+^/PMN^−^ cells at 3 dpi (Figure S2).

For the mRNA extraction and qRT-PCR studies, CD11b^+^/PMN^−^ cells were purified by flow cytometry in a FACSAria^TM^ Cell Sorter (BD Biosciences, San Diego, CA) using BD FACSDiva software. The cells were collected in 100–200 μL extraction buffer from Arcturus Picopure RNA Isolation Kit (12204-01, Applied Biosystems), incubated for 30 min at 42 °C, centrifuged at 800× *g* for 2 min, and the resulting supernatant was collected and stored at −80 °C until use.

### 2.4. mRNA Isolation and Quantitative qRT-PCR

For in vitro gene expression studies, total RNA isolation was carried out using the RNeasy Mini Kit (74104, Qiagen, Madrid, Spain). The RNA concentration and quality were assessed by spectrophotometry (Nanodrop, Thermo Fisher Scientific, Waltham, MA, USA), and subsequently we performed one round of amplification from 250 ng of total RNA, with a MessageAmp II aRNA kit (AM1751, Thermo Fisher Scientific, Madrid, Spain), following manufacturer’s instructions. Next, as previously described [26], 4 µg of total RNA per sample were reverse-transcribed after previous digestion with DNase I (4716728001, Sigma-Aldrich, Steinheim, Germany) to eliminate putative genomic DNA traces. All gene expression analyses were performed in duplicate for each sample, which were obtained from 4 independent cultures.

For ex vivo gene expression analysis in the NL rat spinal cord and after SCI at 24 hpi and at 3, 7, and 14 dpi, total RNA isolation was carried out using the Arcturus Picopure RNA isolation Kit (KIT0204, Thermo Fisher Scientific, Madrid, Spain) following the manufacturer’s instructions. Moreover, to eliminate putative genomic DNA traces, we performed a DNase treatment (79254, Qiagen, Madrid, Spain) directly within the purification column according to the manufacturer’s protocol. For RNA quantity and integrity evaluation, we used an Experion RNA HighSense chip-based electrophoresis kit (7007155 and 7007156, BioRad, Hercules, CA, USA) following the manufacturer’s instructions. Both the quantity and the RNA Quality Indicator (RQI) were suitable (values between 8.2–10.0), so we continued with the next step, consisting in performing an amplification of the RNA that was obtained. To this end, we used a MessageAmp II aRNA kit (AM1751, Thermo Fisher Scientific, Madrid, Spain), and we completed the 2-round amplification protocol following the manufacturer’s instructions. Next, 2 µg of total RNA per sample were reverse-transcribed after previous digestion with DNase I (4716728001, Sigma-Aldrich, Steinheim, Germany). All gene expression analyses were performed in duplicate for each samples (n = 5).

In order to ensure the absence of neutrophil contamination in the spinal cord microglia/MDM-isolated cells, and before the gene expression analysis of the Wnt family members, all the samples were subjects of qRT-PCR to detect the neutrophil elastase enzyme that was encoded by ELANE gene, which is exclusively expressed by these cells [66]. The analysis of ELANE expression and Glyceraldehyde-3-phosphatedehydrogenase (GAPDH) as an endogenous control were performed in 10 ng of total reverse-transcribed RNA with the TaqMan gene expression master mix (4369016, Thermo Fisher Scientific, Madrid, Spain) and specific TaqMan Gene Expression Assays (assay ID: GAPDH: Rn01775763_g1 and ELANE: Rn01535456_g1, Thermo Fisher Scientific, Madrid, Spain). The reactions were run on an ABI PRISM7900HT Fast Sequence Detection System (Applied Biosystems). All gene expression analyses were performed in duplicate for each sample and the cycle threshold (Ct) values above 35 were considered as undetectable. We did not detect ELANE gene expression in any of the analyzed samples.

Both in vitro and ex vivo gene expression analysis of the different Wnt ligands, receptors, and regulators were performed using customized Taq-Man Array Microfluidic Cards (4342253, Thermo Fisher Scientific, Madrid, Spain) and the TaqMan Gene Expression Master Mix (4369016, Thermo Fisher Scientific, Madrid, Spain) in 21 ng of total reverse-transcribed RNA per spot. TaqMan Gene Expression Assays for the different Wnt family genes were the same described in a previous report [59]. GAPDH was used as an endogenous control, and all the reactions were run on an ABI PRISM7900HT Fast Sequence Detection System (Applied Biosystems). A Ct >35 was considered as undetectable. The relative quantification for each gene was performed by the ΔΔCt method [67]. The ΔCt values were calculated as follows: ΔCt = Ct (reference gene)–Ct (gene of interest). Then, the ΔCt value of the control sample (NA or NL) was subtracted from the ΔCt value of the studied sample (treatment or time post-injury) to get the ΔΔCt value. Finally, the relative fold gene expression values were subsequently obtained through 2^ΔΔCt^, and the data were presented as the fold change compared to the NA or NL control group, respectively.

### 2.5. Statistical Analysis

The data are presented as the mean ± SEM. Statistical significance between the groups and times post-activation/post-injury in ΔCt values were determined by one-way ANOVA followed by Bonferroni post hoc test using GraphPad Prism 6 software. A *p* ≤ 0.05 was considered statistically significant.

## 3. Results

### 3.1. Differential Expression of Wnt Family Genes in Intact and Injured Rat Spinal Cord Isolated Microglia/MDM Cells

The first aim of this study was to evaluate the Wnt signaling transcriptome in microglia/MDM cells from rat spinal cord at different times post-injury, that were obtained through the combination of FACS direct isolation followed by mRNA extraction and qRT-PCR analysis. Remarkably, only a few Wnt family members were detected in the isolated microglia/MDM cells. More specifically, we only found detectable gene expression levels in at least at one of the time-points that were analyzed for *Wnt4* and *Wnt5b* ligands, *Fz1*, *Fz8*, *Fz9*, *Ryk,* and *Ptk7* receptors, and *Rspo1*, *sFRP1*, *sFRP4*, *sFRP5,* and *Wif1* modulators (Table 1). We did not find detectable expression levels for *Wnt1*, *Wnt2*, *Wnt2b*, *Wnt3*, *Wnt3a*, *Wnt5a*, *Wnt6*, *Wnt7a*, *Wnt7b*, *Wnt8a*, *Wnt8b*, *Wnt9a*, *Wnt9b*, *Wnt10a*, *Wnt10b*, *Wnt11,* and *Wnt16* ligands, nor for *Fz2*, *Fz3*, *Fz4*, *Fz6*, *Fz7*, *Fz10*, *Ror1,* and *Ror2* receptors, and neither for *Dkk1*, *Dkk2*, *Dkk3*, *Dkk4*, *sFRP2*, *Rspo2,* and *Rspo3* modulators. We found that the higher expression levels were detected for *Fz8* and *Ryk* genes at all time-points that were analyzed, as well as for *Rspo1* from 3 dpi onwards. On the contrary, the lowest expression levels were observed for genes such as *Wnt5b*, *Wif1*, *sFRP4,* and *sFRP5*, which even reached undetectable expression levels for most of the time-points that were analyzed. Finally, due to detection problems with the spots corresponding to *Fz5* and *sFRP3* genes, the number of replicates was reduced to n = 2 in the NL subjects also showing a high variability between samples, and therefore, we cannot obtain conclusive results regarding expression alterations of these specific genes (Table 1).

**Figure 1:**
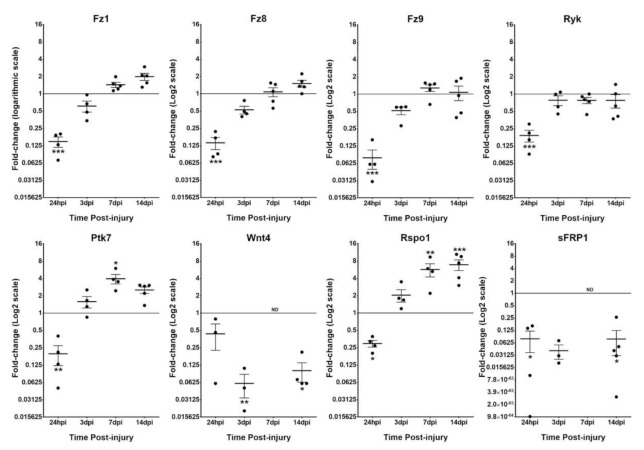
mRNA expression analysis of Wnt-related molecules in spinal cord-isolated microglia at different times post-injury and compared to uninjured conditions. The figure shows mRNA expression data corresponding to different detectable Wnt signaling-related molecules in spinal cord isolated microglial cells at different times post-injury and compared to microglia that were isolated for the intact spinal cord. Data represent 2^ΔΔCt^ mean ± SEM. * *p* < 0.05; ** *p* < 0.01; *** *p* < 0.001. h: hours; d: days; ND: non-detected.

When we compared the relative expression of each detected gene along the different time-points that were analyzed with their respective expression in NL group, we observed that almost all genes, including *Fz1*, *Fz8*, *Fz9*, *Ryk*, *Ptk7*, *Rspo1,* and *sFRP1* were significantly downregulated at 24 hpi (Figure 1). Thereupon, the expression seems to return to basal levels in case of *Fz1*, *Fz8*, *Fz9,* and *Ryk*, whereas we found an interesting switch in the expression of *Ptk7* at 7 dpi and of *Rspo1* at 7–14 dpi, increasing their expression levels significantly at these time-points (Figure 1). Moreover, the expression levels of *sFRP1* and *Wnt4* remained downregulated along all times post-injury (even undetectable at 7 dpi).

### 3.2. Differential Expression of Wnt Family Genes in Rat Non-Activated and Activated Microglial Cultured Cells When Exposed to a 24 hpi-SCE Versus a Standard LPS Protocol of Stimulation

Next, we aimed to determine whether the stimulation of primary rat microglial cell cultures would reproduce the Wnt transcriptome of FACS isolated/MDM cells that were analyzed above. To this end we have used two different stimulatory systems: a prototypical LPS inflammatory activation and the 24 hpi SCE activation (Table 2). We did not find detectable expression levels under any experimental condition for *Wnt2*, *Wnt5a*, *Wnt6*, *Wnt7a*, *Wnt7b*, *Wnt9a*, *Wnt10a*, *Wnt10b*, *Wnt11,* and *Wnt16* ligands, nor for *Fz4* and *Ror1* receptors, and neither for *Dkk4* and *Rspo2* modulators.

#### 3.2.1. Wnt Ligands

Regarding Wnt ligands, when compared to NA microglial cells both LPS- and SCE-mediated stimulation led to a lack of significant changes in the expression of *Wnt4*, *Wnt6*, *Wnt7a*, *Wnt7b*, *Wnt9a,* and *Wnt11* at 24 hpa, and of *Wnt7a* at 3 dpa. Moreover, and again independently of the activation system that was used, *Wnt7b* and *Wnt10a* strongly decreased their expression at 3 dpa until they reached undetectable levels while both activation systems also downregulated *Wnt1* and *Wnt16* expression at 24 hpa.

**Figure 2:**
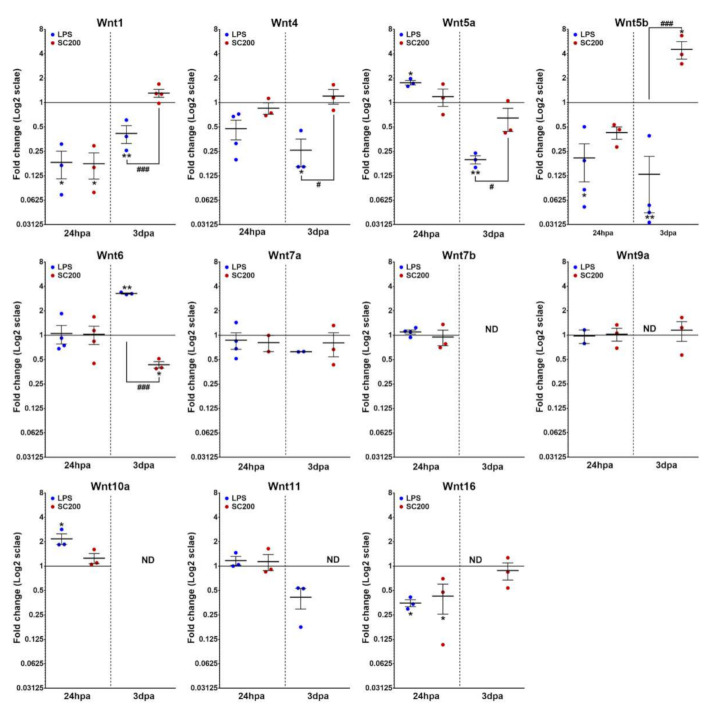
mRNA expression analysis of Wnt ligands in LPS- or SCE-activated microglia and compared to non-activated basal conditions. The figure shows mRNA expression data corresponding to different detectable Wnt ligands in activated microglial cells either with 100 ng/mL of lipopolysaccharide (LPS) or with 200 µg/mL of lesioned spinal cord extract (SCE). Data represent 2^ΔΔCt^ mean ± SEM. * indicate differences between treated cells vs. non-activated. # indicate differences between treatments (LPS vs. SCE). * *p* < 0.05; ** *p* < 0.01; *** *p* < 0.001; # *p* < 0.05; ## *p* < 0.01; ### *p* < 0.001. hpa: hours post-activation; dpa: days post-activation.

By contrast, we observed some post-activation changes that clearly depended on the activation system that was used. On the one hand, LPS-activation led to the downregulation of *Wnt5b* at 24 hpa, and *Wnt1*, *Wnt4*, *Wnt5a,* and *Wnt5b* at 3 dpa. Conversely, LPS-activation induced an increase in *Wnt5a* and *Wnt10a* expression at 24 hpa, and in *Wnt6* at 3 dpa, whereas we observed no significant changes in *Wnt11* expression at 3 dpa. Moreover, we did not find detectable levels of expression in LPS-activated cells for *Wnt9a* and *Wnt16* genes at 3 dpa. On the other hand, SCE-mediated activation led to a decreased expression of only *Wnt6* at 3 dpa. However, the same activation system induced the upregulation of only *Wnt5b* at 3 dpa. No significant changes were found for *Wnt5a*, *Wnt5b,* and *Wnt10a* gene expression at 24 hpa, neither for *Wnt1*, *Wnt4*, *Wnt5a*, *Wnt9a,* and *Wnt16* at 3 dpa. Finally, we did not find detectable levels of expression in SCE-activated cells for *Wnt11* at 3 dpa (Figure 2).

#### 3.2.2. Wnt Receptors

A subset of receptor genes showed no significant changes in their expression pattern when compared with NA state independently of the activation system that was used, such as *Fz5*, *Fz6*, *Fz9,* and *Fz10* at 24 hpa, and as *Ryk* at 3 dpa. Besides, both LPS- and SCE-mediated activation led to the downregulation of *Fz1* expression at 24 hpa.

**Figure 3:**
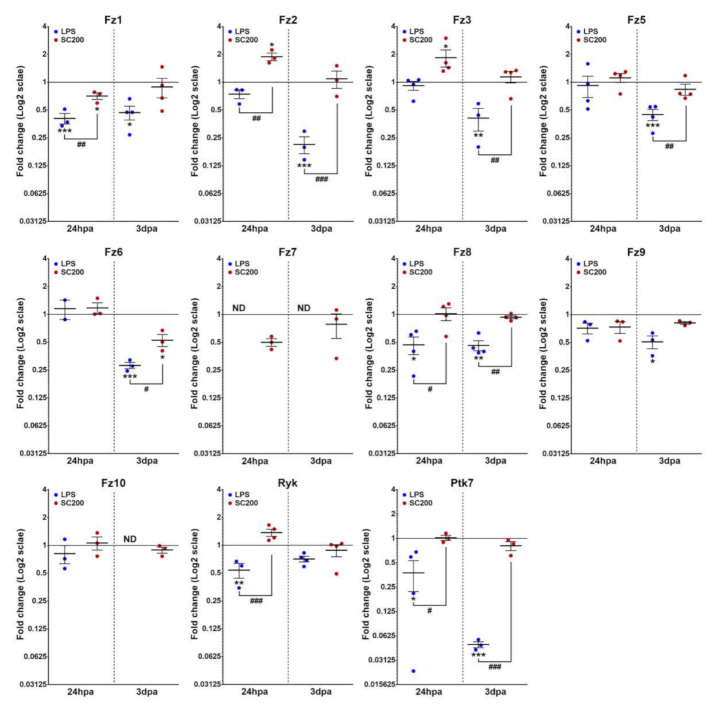
mRNA expression analysis of Wnt receptors in LPS- or SCE-activated microglia and compared to non-activated basal conditions. The figure shows mRNA expression data corresponding to different detectable receptors in activated microglial cells either with 100 ng/mL of lipopolysaccharide (LPS) or with 200 µg/mL of lesioned spinal cord extract (SCE). Data represent 2^ΔΔCt^ mean ± SEM. * indicate differences between treated cells vs. non-activated. # indicate differences between treatments (LPS vs. SCE). * *p* < 0.05; ** *p* < 0.01; *** *p* < 0.001; # *p* < 0.05; ## *p* < 0.01; ### *p* < 0.001. hpa: hours post-activation; dpa: days post-activation.

However, various post-activation changes that clearly depended on the activation system that was used were observed again. More specifically, LPS-activation led to the downregulation of *Fz8*, *Ryk,* and *Ptk7* expression at 24 hpa, and *of Fz1*, *Fz2*, *Fz3*, *Fz5*, *Fz6*, *Fz8*, *Fz9,* and *Ptk7* at 3 dpa. Nevertheless, we observed no significant changes in *Fz2* and *Fz3* expression at 24 hpa. We did not find detectable levels of expression in the LPS-activated cells for *Fz7* at 24 hpa and for *Fz7* and *Fz10* at 3 dpa. Instead, SCE-mediated activation led to a decrease of *Fz6* expression at 3 dpa. However, the same activation system induced the upregulation of *Fz2* and *Fz3* expression at 24 hpa, Lastly, no significant changes were found for *Fz7*, *Fz8*, *Ryk,* and *Ptk7* gene expression at 24 hpa, neither for *Fz1*, *Fz2*, *Fz3*, *Fz5*, *Fz7*, *Fz8*, *Fz9*, *Fz10,* and *Ptk7* at 3 dpa (Figure 3).

#### 3.2.3. Wnt Modulators

Independent of the activation system that was applied, several Wnt-related modulators genes showed no significant changes in their expression pattern when compared with NA state, such as *Rspo3*, *sFRP1,* and *sFRP3* at 24 hpa. Moreover, both LPS- and SCE-mediated activation led to the downregulated expression of the Wnt-related modulators *Dkk1* and *Wif1* at 3 dpa. Additionally, *Rspo3* strongly decreased its expression at 3 dpa until it reached undetectable levels independently of the activation system that was applied.

**Figure 4:**
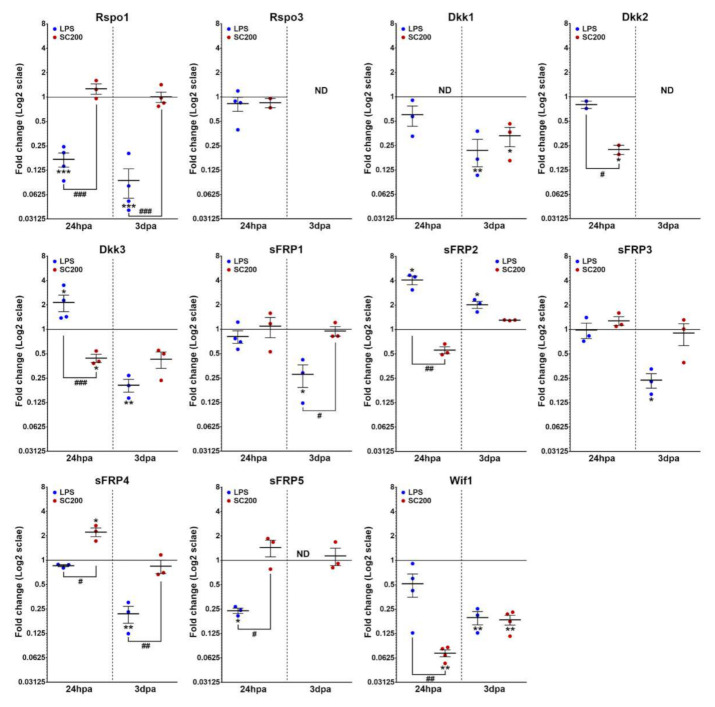
mRNA expression analysis of Wnt inhibitors and modulators in LPS- or SCE-activated microglia and compared to non-activated basal conditions. The figure shows mRNA expression data corresponding to different detectable Wnt signaling inhibitors and modulators in activated microglial cells either with 100 ng/mL of lipopolysaccharide (LPS) or with 200 µg/mL of lesioned spinal cord extract (SCE). Data represent 2ΔΔCt mean ± SEM. * indicate differences between treated cells vs. non-activated. # indicate differences between treatments (LPS vs. SCE). * *p* < 0.05; ** *p* < 0.01; *** *p* < 0.001; # *p* < 0.05; ## *p* < 0.01; ### *p* < 0.001. hpa: hours post-activation; dpa: days post-activation.

Although, we also observed several post-activation changes that clearly depended on the activation system that was used. On the one hand, LPS-activation led to the downregulation of *Rspo1* and *sFRP5* genes at 24 hpa, and of *Rspo1*, *Dkk3*, *sFRP1*, *sFRP3,* and *sFRP4* at 3 dpa. Conversely, the same activation system induced an increase in *Dkk3* and *sFRP2* gene expression at 24 hpa, and in *sFRP2* at 3 dpa, but we observed no significant changes in *Dkk1*, *Dkk2*, *sFRP4,* and *Wif1* expression at 24 hpa. We did not find detectable levels of expression in the LPS-activated cells for *sFRP5* at 3 dpa. On the other hand, SCE-mediated activation led to a decreased expression of *Dkk2*, *Dkk3,* and *Wif1* at 24 hpa. However, the same activation system induced the *sFRP4* upregulation at 24 hpa. No significant changes were found for *Rspo1*, *sFRP2,* and *sFRP5* gene expression at 24 hpa, neither for *Rspo1*, *Dkk3*, *sFRP1*, *sFRP2*, *sFRP3*, *sFRP4,* and *sFRP5* at 3 dpa. Finally, we did not find detectable levels of expression in SCE-activated cells for *Dkk1* at 24 hpa (Figure 4).

### 3.3. Differential Expression of Wnt Family Genes by Microglia/MDM Cells Depending on the Experimental Approach (Ex Vivo or In Vitro) Used

**Figure 5:**
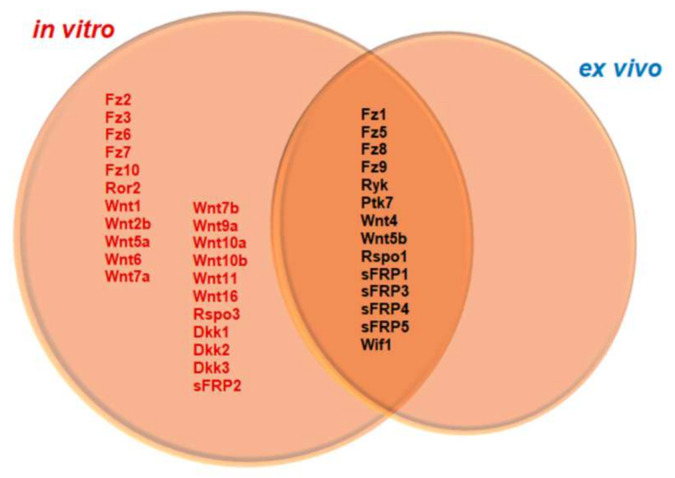
Differential gene expression of Wnt-related molecules among non-activated microglia in vitro and isolated microglia from uninjured spinal cord. Venn diagram shows Wnt-related genes that were detected both in vitro and ex vivo basal conditions. Text in red identify those genes exclusively detected in non-activated microglial cells in vitro, whereas the text in black identifies those genes that were detected in both non-activated cells in vitro and microglia cells that were isolated from uninjured spinal cord. There is no gene exclusively that was detected in the uninjured spinal cord microglia.

**Figure 6:**
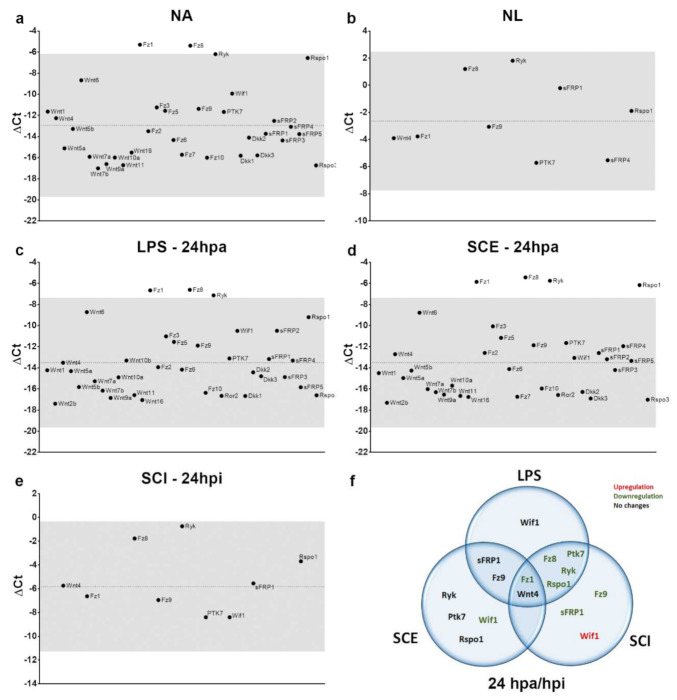
Schematic overview of harmful stimulus effect on microglial cells both in vitro and ex vivo for the three experimental conditions that were evaluated at 24 h post-treatment/injury. Graphic representation of ΔCt values for the different genes that were analyzed in the different evaluated conditions, where the dashed line indicates ΔCt mean value in each case and gray shading identifies an area corresponding to the mean value ± 2 times the standard deviation to highlight those genes with higher/lower expression levels for each experimental condition. (**a**) Wnt-related genes that were detected in non-activated microglial cells in vitro, (**b**) Wnt-related genes that were detected in microglial cells isolated from non-lesioned spinal cord, (**c**) Wnt-related genes that were detected in LPS-activated microglial cells in vitro at 24 hpa, (**d**) Wnt-related genes that were detected in SCE-activated microglial cells in vitro at 24 hpa, (**e**) Wnt-related genes that were detected in microglial cells that were isolated from injured spinal cord at 24 hpi, (**f**) Venn diagram shows individual and shared gene alterations that were observed for the three experimental conditions that were evaluated at 24 h post-activation/injury. NA: non-activated; NL: non-lesioned; LPS: lipopolysaccharide; SCE: spinal cord extract; SCI: spinal cord injury; hpa: hours post-activation; dpa: days post-activation; hpi: hours post-injury.

Finally, we analyzed the parallelisms and discrepancies that we had found in the Wnt transcriptome between the ex vivo and in vitro experimental approaches that were applied. As we illustrate in Figure 5, a comparison of Wnt-related genes exhibiting detectable expression under basal conditions both in vitro (NA) and ex vivo (NL) showed that, although both in vitro cultured microglia and ex vivo isolated microglial cells shared the expression of several Wnt-related genes (genes in black), the bulk of the genes that were detected were only found in cultured microglia (genes in red and black). Conversely, we did not find any gene that was exclusively detected in the uninjured spinal cord. Moreover, when we examined the expression levels (ΔCt) globally we observed that, in control conditions (NA and NL) as well as at 24 h post-treatment/injury, both in vitro and ex vivo, there were always two genes that showed higher expression levels or were found among the genes that were most actively expressed: *Fz8* and *Ryk*. (Figure 6a–e). Furthermore, NA and activated cultured cells also exhibited high expression levels of *Fz1* (Figure 6a,c,d), whereas this gene was not among the most expressed within the spinal cord-isolated microglial cells from NL and 24 hpi (Figure 6b,e). Strikingly, when we focus on the gene expression pattern that was detected at 24 hpa for the LPS/SCE-treated cells and for SCI-isolated cells at 24 hpi, we only observed similar alterations for *Wnt4* and *Fz1* genes (Figure 6f). On the one hand, some genes showed no changes between the three study conditions (LPS, SCE, or SCI), such as *Wnt4*, or between LPS- and SCE-treated cells, as *sFRP1* and *Fz9*. However, microglial cells that were activated with LPS and isolated from SCI shared the downregulation of *Rspo1*, *Ryk*, *Ptk7*, *Fz8,* and *Fz1* genes, being the latter also downregulated in the SCE-activated cells. On the other hand, we also observed several genes whose expression pattern differed depending on the condition/treatment of the cells. The most intriguing situation was observed in case of the *Wif1* gene, whose expression was unchanged in LPS-treated cells but downregulated in the SCE-treated group, whereas the isolated microglial cells from SCI showed an upregulation of the gene. Other genes such as *Ryk*, *Ptk7,* or *Rspo1* differ in their expression pattern based on the treatment of the cells (LPS or SCE) and also between the SCE-treated cells and the SCI-isolated cells.

## 4. Discussion

Microglia are the resident immune cells in the CNS that are responsible for both the maintenance of brain and spinal cord tissue homeostasis, as well as for the safeguard of neural cells from either intrinsic or extrinsic insults [1,68]. In the context of an SCI, the initial mechanical trauma elicits a robust inflammatory response that is mediated by resident microglia activation and the subsequent infiltration of different immune cell populations, including bloodborne monocytes which differentiate into MDMs and remain at/nearby the injury site for months post-injury [1,6,69,70]. These early microglial/MDMs response has both beneficial and harmful effects which critically influence the development and outcome of this neuropathology [1,6,11,13,14,15,16].

Further understanding of molecular signaling pathways that are involved in microglia/MDMs cellular responses to traumatic injuries may help us to pinpoint future therapeutic targets that promote the beneficial effects that are regulated by these cells while preventing the detrimental and neurotoxic ones. Interestingly, it is now increasingly evident that the Wnt family of proteins is involved in different neuropathologies that are characterized by a dysregulated neuroinflammatory response [17,18,19,20], including SCI [21,22,23,24,25,26,27,28,29,30,31]. As a consequence, we decided to assess whether the use of ex vivo and in vitro methods might help to foster the characterization of the gene expression alterations of Wnt-related molecules of microglia/MDMs cells in the traumatically-lesioned rat spinal cord. Multiple protocols have been designed to purify by FACS or enrich by immunopanning or magnetic sorting microglia/MDM cells to analyze their transcriptome and maintain in cell culture [55]. However, most of them have been performed on human and mouse microglia, while the most common animal model that is used in SCI research is the rat (72.4%) [63]. Noteworthy, significant differences on microglia/MDM cell expression patterns and response to the same stimulus have been described between human and rodents, as well as mice and rats [56,57,71]. Following this rationale, efforts to specifically analyze rat microglia/MDM cells and mimic in vitro the SCI pathophysiology have been lately made [56,59,60,65].

First of all, we aimed to characterize the Wnt transcriptome of microglia/MDM cells at different time-points after contusive SCI in order to obtain a first ex vivo reference of the global Wnt pattern of expression that is elicited specifically in this cell type and species. Noteworthy, the purification of both microglia and MDM cells has long relied on the expression of constitutive surface markers of the myeloid cell lineage such as CD11b and CD45 [55]. Although single cell transcriptomic studies have allowed the identification of several microglia-specific markers [72,73], which have been extensively used for the generation of transgenic mice to specifically analyze the specific roles of microglia cells in CNS damage [4], not all these markers are equally represented in mice, human, and rats microglia cells [4,16,71,72,74]. Interestingly, Iba1 and low/high expression of CD45 has been recently claimed as able to discriminate microglia from MDM and neutrophil cells in a seven days post-contusion injured rat spinal cord [60]. Therefore, we decided to first assess the common combination of CD11b with low/high CD45 expression to differentiate microglia from MDM and neutrophil cells in our experimental design, implemented by using a PMN antibody that was already validated [65] to further eliminate the second richest CD11b^+^ and CD45^+^ neutrophil population from our transcriptomic analysis. However, we were able to discern a CD45-low expressing microglia from CD45-high expressing MDM cells at only three days out of a time course including 1, 3, 7, and 14 days-post-contusion (Figure S2). Our results seem to support other experimental evidences questioning CD45 as a reliable marker to discriminate microglia from MDM cells, which has been shown to increase in specifically spinal microglia cells after peripheral nerve damage in rats and after SCI in mice [75]. Furthermore, and in agreement with our results, an interesting study of Noristani et al. by using a combination of CD11b, CD45, and Ly6C to differentiate microglia and macrophage cells in transgenic mice that were overexpressing eGFP under the control of the CX3CR1 promoter, found two clearly differentiated low and high CD45 subpopulations at 3 but not 7 or 14 days after SCI [76]. Therefore, we decided to exclude CD45 sorting from the final protocol and analyze both microglia and MDM cells as a single population. It should be noted that the incapacity to differentiate microglia from MDM cells in the present study might be underlying some of the differences in the Wnt expression pattern that was observed since, as previously described [4,15,58], the ratio of microglia/MDM suffer evident changes during the temporal progression of SCI. Interestingly, in a recent work that was performed in mice, the authors analyzed microglia and macrophage transcriptomes separately by single-cell RNA sequencing at five days after SCI and, regarding Wnt-family members genes, it was not found a significantly different expression between these two cell populations [77]. Hence, future studies must address the competence to identify the microglia- and MDM-specific Wnt expression patterns, in order to pave the way for further research that is addressed to determine their contribution to cell-type-specific roles or phenotypes of activation.

In our study, we only detected gene expression of a few Wnt-related genes on isolated microglia/MDMs from uninjured spinal cord, *Fz8* and *Ryk* being the most expressed. Noteworthy, we observed that almost all the genes that were detected were significantly downregulated in the first 24 hpi (Figure 1). Interestingly, there are several previous studies regarding transcriptomic changes in microglia/MDM cells in mice experimental models of SCI, from which some information concerning Wnt-related gene expression could be deciphered [76,77,78,79]. For example, Wnt ligands seems to be less expressed within the Wnt transcriptome of microglia/MDM cells compared to the receptors, and also some modulators such as Dkk1, Dkk2, Dkk4, or sFRP2 appear to not be detected across these studies. However, different models of SCI, including hemisection, transection, and contusion injury have been used for these studies, and also different transgenic mice strains and single-cell or bulk RNA sequencing approaches to generate a specific microglia transcriptomics were used. Altogether, all these factors make it extremely difficult to draw reliable conclusions or to establish well founded comparisons between our results and the bibliography. To our knowledge, these are the first evidence of Wnt-related gene expression in microglia/MDM cells that are isolated from intact and injured rat spinal cord by using the ex vivo approach that is described here, which might suppose a closer reflection of physiological and pathological conditions in the rat spinal cord.

On the other hand, we sought for an in vitro model of neuroinflammation that is able to mimic the pathophysiology of SCI in order to generate a potential method for future assessing of whether different patterns of expression of the Wnt family or their modulation would contribute to elicit an anti-inflammatory response. Widely-used experimental approaches include primary cultures of neonatal and murine microglia cell lines such as BV2, and their stimulation with LPS, pro-inflammatory cytokines, or protein extracts from injured spinal cords [56,60,80]. Despite of its low yield and being time-consuming, the culture of primary neonatal microglia is the closest to adult microglia that are currently available [80]. Moreover, although LPS has been the most extensive method used to elicit the so-called M1 or pro-inflammatory state of microglial cells, there is increasing evidence that evokes an overwhelming mixed pro- and anti-inflammatory response that is rather different than the one that is observed with pro-inflammatory cytokines or a spinal cord extract [56,60]. Interestingly, the exposure of primary neonatal rat microglial cells to 50 µg/mL of a 7 dpi SCE allowed to mimic the pathophysiological conditions that are found in vivo and elicit resembling M1 and M2 active phenotypes, including an increase of area, proliferation, phagocytosis, and a balanced expression of pro- and anti-inflammatory cytokines [56,60]. Following the same rationale, we used an SCE that was obtained from spinal cords at 24 h after performing the same contusion injury as for the ex vivo study, since the peak of expression of pro-inflammatory cytokines and the onset of microglial activation occurs in the first 24 hpi [11,12]. Furthermore, when compared to the oversized inflammatory response that is elicited by LPS, the Wnt expression pattern was also partially different and this could be due to a more physiological endogenous response that is elicited by SCE stimulation on microglia/MDM cells compared to LPS, since SCI injury concurs with a complex and variable post-injury cocktail of cytokines and damage-associated molecules as the main triggers of microglial activation [4,5,56,58]. Future studies should extend on correlating these Wnt expression profiles to the different microglial states of activation, in order to settle the basis to characterize the specific roles and therapeutic potential of each Wnt protein on the inflammatory response and modulation of microglial cells.

Interestingly, our results are complementary to previous reports [32,33], and we found that non-activated microglial cultured cells expressed most of the Wnt-related molecules, including ligands, receptors, and soluble modulators. However, when we analyzed the effect that is induced by each stimulation method, we observed that similarities in Wnt-related genes alterations were really scarce, being even detected as opposite responses for some of the genes depending on the stimulatory agent, such as *Dkk3* at 24 hpa or *Wnt5b* and *Wnt6* at 3 dpa. Moreover, when we examined the expression levels (ΔCt) globally we observed that in control conditions (NA and NL) as well as at 24 h post-treatment, there were two genes that were the most actively expressed: *Fz8* and *Ryk*, coinciding with what we observed ex vivo.

In this context, different studies have shown in the past that primary microglia and microglia cell lines are able to respond to Wnt signals since these cells express several Fz receptors and co-receptors [32,33]. However, most of the existing works are mainly focused on Wnt canonical signaling activation/inhibition in microglial and macrophage cells, and how β-catenin levels are altered on these activated cells. Intriguingly, different evidence has been found concerning the pro- or anti-inflammatory microglial response that is modulated by β-catenin-dependent signaling. On the one hand, different evidence both in vitro and in vivo point to an anti-inflammatory phenotype of microglial cells that is promoted by Wnt/β-catenin signaling activation, whereas the Wnt/β-catenin pathway inhibition seems to drive a pro-inflammatory phenotypic transformation of these cells [34,35,36,40,44]. On the other hand, other studies suggest that the downregulation of Wnt/β-catenin signaling attenuates local inflammation and reduces inflammatory cytokine release from microglia/MDMs, while Wnt/β-catenin signaling activation by, for example Wnt3a, induces a pro-inflammatory transformation [21,33,37,38,42,43]. Furthermore, there is also some evidence that involves β-catenin-independent signaling that is mediated by Wnt5a with a pro-inflammatory transformation of microglial cells [32,81]. Remarkably, depending on the cellular or physiological context, the same Wnt molecule may either promote or suppress microglial/MDMs activation, acting as both a pro- and anti-inflammatory regulator. It has been described that both the canonical Wnt3a and the non-canonical Wnt5a ligands lead to a pro-inflammatory response in cultured primary mouse microglia [32,33,42]. However, LPS pre-activated microglia exhibit a dose-dependent decrease in pro-inflammatory markers when treated with Wnt3a and Wnt5a, suggesting a dual role of Wnt pathways on microglia regulation in a context-dependent manner [41]. Therefore, while there is some evidence of the Wnt signaling involvement in the inflammatory response of microglia/MDMs, there are limited data on how specific noxious stimuli which lead to an inflammatory response influence or impact the Wnt signaling-related molecules expression patterns on these cells.

Strikingly, as we have mentioned before, it is well known that microglia are able to respond to Wnt signals but, at present, little is known regarding microglial cells as a potential source of Wnt ligands and related soluble modulators. For instance, it has been described that the secretion of Wnt proteins by microglial cells in vitro depends on the activation state of these cells [82]. The authors showed how M1 polarized microglia released Wnt5a while on the contrary, M2c polarized microglia secreted Wnt7a. Furthermore, Wnt7a conditioned media that was secreted by M2c microglia promoted oligodendrogenesis in neural stem cells cultures [82]. The scarce evidence points not only to the potential modulatory role of Wnt signals on microglial cells in the inflammatory response, but also in the influence of Wnt signals that are released by microglia in the surrounding environment and neighbor cells, but further studies are needed to shed light on this issue.

In this context, it should be noted that it has been previously documented that primary cultured microglia, as well as different widely used microglial cell lines, display drastic differences in their gene expression profile and behavior compared to those that are immediately isolated from tissue [83,84]. It has been described that microglial isolation from their tissue context induces a prominent activation profile on these cells [85,86]. Furthermore, primary microglial cultures are commonly prepared from neonatal brain, and may not reliably reflect the real state of microglia in the adult or aged individual [87]. All these issues raise questions about whether in vitro data regarding microglia can be reliably compared to their in vivo counterparts. For instance, some authors have reported that primary cultured mice microglial cells as well as the N13 microglial cell line express many Fzs receptors in vitro, although they exhibited some differences between their expression patterns [33]. In this line, we have shown in a previous study that Ryk receptor was not detected in microglial cells of the adult rat spinal cord, whereas we observed a clear induction of Ryk expression in many activated microglia/macrophage cells from 3 dpi on in the lesioned areas of contusioned rats [28]. Conversely, here we observed no significant changes of Ryk expression by primary cultured microglial cells that were stimulated with SCE neither at 24 hpa or 3 dpa, while isolated microglia from lesioned spinal cord exhibited a significant decrease of its expression at 24 hpi compared to intact animals.

Taken together, our results indicate that the expression profile of Wnt-related genes in microglial cells that are cultivated in vitro, as well as isolated ex vivo and those that are analyzed in its tissular environment in vivo exhibit important differences, which would be in line with previous studies where similar discrepancies have been described for other molecules. While the in vitro systems play a key role for broadening our knowledge for the functioning and behavior of these cells, it is also clear that their heterogeneity, their region-specific differences, and the complexity on their tissue interactions with other cells will also need to be considered. Accordingly, our study points to the need to pursue the search for experimental models for studying microglial cells behavior in physiological and pathological conditions that are capable of achieving results that are more likely to be clinically translational. In spite of that, we believe that our results provide a first experimental approach that is designed to specifically characterize the Wnt expression pattern and roles in microglia and MDM cells when using a rat model of SCI, which considering all its limitations, we expect will contribute to foster the research on Wnt-driven immunomodulatory therapies.

## Figures and Tables

**Table 1:** ΔCts values ± SEM of all the detected genes at the different times post-injury that were evaluated. NL: non-lesioned; hpi: hours post-injury; dpi: days post-injury; ND: non detected; ?: inconclusive results.

Gene	NL	24 hpi	3 dpi	7 dpi	14 dpi
Fz1	−3.77 ± 0.51	−6.63 ± 0.34	−4.58 ± 0.32	−3.28 ± 0.14	−2.84 ± 0.20
Fz5	?	−9.31 ± 0.57	−6.05 ± 0.35	−5.71 ± 0.90	−6.63 ± 0.94
Fz8	1.20 ± 0.22	−1.77 ± 0.36	0.22 ± 0.20	1.21 ± 0.28	1.74 ± 0.19
Fz9	−3.06 ± 0.12	−6.95 ± 0.46	−4.08 ± 0.27	−2.78 ± 0.23	−3.24 ± 0.46
Ryk	1.81 ± 0.16	−0.74 ± 0.38	1.37 ± 0.30	1.39 ± 0.21	1.24 ± 0.38
Ptk7	−5.72 ± 0.61	−8.40 ± 0.60	−5.17 ± 0.33	−3.81 ± 0.27	−4.45 ± 0.22
Wnt4	−3.90 ± 0.24	−5.74 ± 1.14	−8.21 ± 0.66	ND	−7.49 ± 0.45
Wnt5b	ND	ND	−7.49 ± 1.02	ND	ND
Rspo1	−1.89 ± 0.41	−3.69 ± 0.20	−0.98 ± 0.32	−0.10 ± 0.63	0.74 ± 0.35
sFRP1	−0.21 ± 0.46	−5.55 ± 1.64	−5.14 ± 0.61	ND	−5.03 ± 1.03
sFRP3	?	?	−6.89 ± 0.31	ND	ND
sFRP4	−5.53 ± 0.69	ND	ND	ND	ND
sFRP5	ND	ND	ND	ND	−6.99 ± 0.58
Wif1	ND	−8.40 ± 0.95	−8.35 ± 0.50	ND	ND

**Table 2:** ΔCts values ± SEM of all detected genes at the different times post-activation that were evaluated and for the three experimental situations that were analyzed. hpa: hours post-activation; dpa: days post-activation; NA: non-activated; ND: non detected; LPS: lipopolysaccharide; SCE: spinal cord extract.

	24 hpa			3 dpa		
Gene	NA	LPS	SCE	NA	LPS	SCE
Fz1	5.30 ± 0.05	−6.66 ± 0.13	−5.85 ± 0.16	−4.11 ± 0.07	−5.27 ± 0.30	−4.40 ± 0.29
Fz2	−13.50 ± 0.12	−13.94 ± 0.19	−12.59 ± 0.22	−11.11 ± 0.28	−13.40 ± 0.19	−11.06 ± 0.12
Fz3	−11.24 ± 0.06	−11.02 ± 0.19	−10.07 ± 0.34	−9.61 ± 0.22	−11.27 ± 0.21	−9.47 ± 0.19
Fz5	−11.56 ± 0.23	−11.54 ± 0.21	−11.16 ± 0.33	−10.90 ± 0.15	−12.11 ± 0.10	−11.19 ± 0.07
Fz6	−14.33 ± 0.30	−14.17 ± 0.35	−14.12 ± 0.18	−11.55 ± 0.14	−13.40 ± 0.11	−12.51 ± 0.21
Fz7	−15.73 ± 0.08	ND	−16.74 ± 0.12	−14.74 ± 0.07	ND	−15.27 ± 0.40
Fz8	−5.39 ± 0.27	−6.61 ± 0.34	−5.43 ± 0.14	−4.73 ± 0.12	−5.88 ± 0.26	−4.84 ± 0.10
Fz9	−11.38 ± 0.17	−11.89 ± 0.32	−11.86 ± 0.35	−11.45 ± 0.26	−12.46 ± 0.08	−11.75 ± 0.28
Fz10	−16.01 ± 0.17	−16.37 ± 0.29	−15.96 ± 0.20	−15.43 ± 0.03	ND	−15.60 ± 0.15
Ryk	−6.19 ± 0.05	−7.13 ± 0.23	−5.75 ± 0.16	−4.09 ± 0.18	−4.60 ± 0.08	−4.34 ± 0.10
Ror2	ND	−16.66 ± 0.48	−16.57 ± 0.62	ND	ND	ND
Ptk7	−11.67 ± 0.26	−13.11 ± 0.32	−11.65 ± 0.36	−7.62 ± 0.30	−11.99 ± 0.34	−7.95 ± 0.49
Wnt1	−11.64 ± 0.24	−14.23 ± 0.80	−14.50 ± 0.47	−14.06 ± 0.06	−15.39 ± 0.23	−13.70 ± 0.18
Wnt2b	ND	−17.40 ± 0.20	−17.31 ± 0.11	ND	ND	ND
Wnt4	−12.26 ± 0.27	−13.52 ± 0.56	−12.71 ± 0.18	−13.58 ± 0.16	−15.71 ± 0.56	−13.37 ± 0.38
Wnt5a	−15.12 ± 0.16	−14.31 ± 0.09	−14.97 ± 0.22	−12.90 ± 0.12	−15.26 ± 0.14	−13.67 ± 0.52
Wnt5b	−13.28 ± 0.18	−15.82 ± 0.66	−14.26 ± 0.14	−11.88 ± 0.21	−15.62 ± 0.69	−9.63 ± 0.11
Wnt6	−8.67 ± 0.33	−8.72 ± 0.49	−8.78 ± 0.35	−11.06 ± 0.14	−9.35 ± 0.12	−12.28 ± 0.26
Wnt7a	−15.92 ± 0.50	−15.27 ± 1.03	−16.02 ± 0.08	−14.58 ± 0.29	−15.77 ± 0.23	−13.66 ± 0.96
Wnt7b	−17.02 ± 0.17	−16.16 ± 0.66	−16.30 ± 0.75	ND	ND	ND
Wnt9a	−16.61 ± 0.25	−16.85 ± 0.10	−16.54 ± 0.47	−15.27 ± 0.25	ND	−15.36 ± 0.45
Wnt10a	−15.99 ± 0.12	−14.90 ± 0.26	−15.69 ± 0.24	ND	ND	ND
Wnt10b	ND	−13.31 ± 1.34	ND	−11.39 ± 0.18	−13.12 ± 0.33	−13.48 ± 0.33
Wnt11	−16.72 ± 0.18	−16.58 ± 0.26	−16.66 ± 0.35	−15.49 ± 0.11	−16.37 ± 0.10	ND
Wnt16	−15.52 ± 0.06	−17.05 ± 0.11	−16.76 ± 0.37	−15.24 ± 0.13	ND	−15.59 ± 0.30
Rspo1	−6.56 ± 0.25	−9.20 ± 0.15	−6.16 ± 0.44	−3.52 ± 0.15	−7.52 ± 0.25	−3.84 ± 0.40
Rspo3	−16.74 ± 0.04	−16.60 ± 0.66	−17.02 ± 0.23	−15.45 ± 0.11	ND	ND
Dkk1	−15.82 ± 0.14	−16.67 ± 0.35	ND	−13.32 ± 0.26	−15.71 ± 0.30	−15.04 ± 0.32
Dkk2	−14.11 ± 0.22	−14.43 ± 0.15	−16.28 ± 0.18	ND	ND	ND
Dkk3	−15.79 ± 0.13	−14.80 ± 0.28	−16.90 ± 0.27	−12.91 ± 0.22	−15.24 ± 0.45	−14.23 ± 0.28
sFRP1	−13.74 ± 0.20	−13.17 ± 0.94	−12.60 ± 1.52	−12.29 ± 0.16	−14.31 ± 0.35	−12.38 ± 0.35
sFRP2	−12.52 ± 0.23	−10.49 ± 0.51	−13.19 ± 0.32	−10.58 ± 0.17	−9.73 ± 0.17	−10.07 ± 0.17
sFRP3	−14.37 ± 0.19	−14.88 ± 0.45	−14.21 ± 0.12	−14.21 ± 0.12	−16.34 ± 0.30	−14.52 ± 0.65
sFRP4	−13.08 ± 0.19	−13.31 ± 0.15	−11.95 ± 0.37	−10.79 ± 0.12	−13.07 ± 0.29	−11.08 ± 0.37
sFRP5	−13.77 ± 0.27	−15.84 ± 0.27	−13.34 ± 0.54	−13.42 ± 0.01	ND	−13.79 ± 0.17
Wif1	−9.93 ± 0.09	−10.50 ± 0.32	−13.06 ± 0.56	−6.67 ± 0.11	−8.82 ± 0.07	−8.91 ± 0.37

## Supplementary Materials

The following supporting information can be downloaded at: https://www.mdpi.com/article/10.3390/brainsci12060708/s1, Figure S1: Microscopic analysis of microglia cell cultures; Figure S2: Gating strategy for rat microglia isolated by fluorescent activated cell sorting (FACS).
