# Supplementary material for: Characterization of Ex Vivo and In Vitro Wnt Transcriptome Induced by Spinal Cord Injury in Rat Microglial Cells
Source: Brain Sci. 2022 May 30;12(6):708. doi: 10.3390/brainsci12060708 (PMC9221341; doi:10.3390/brainsci12060708)
Supplement: Supplementary file 1 [file brainsci-12-00708-s001.zip › brainsci-1648811-supplementary.pdf]

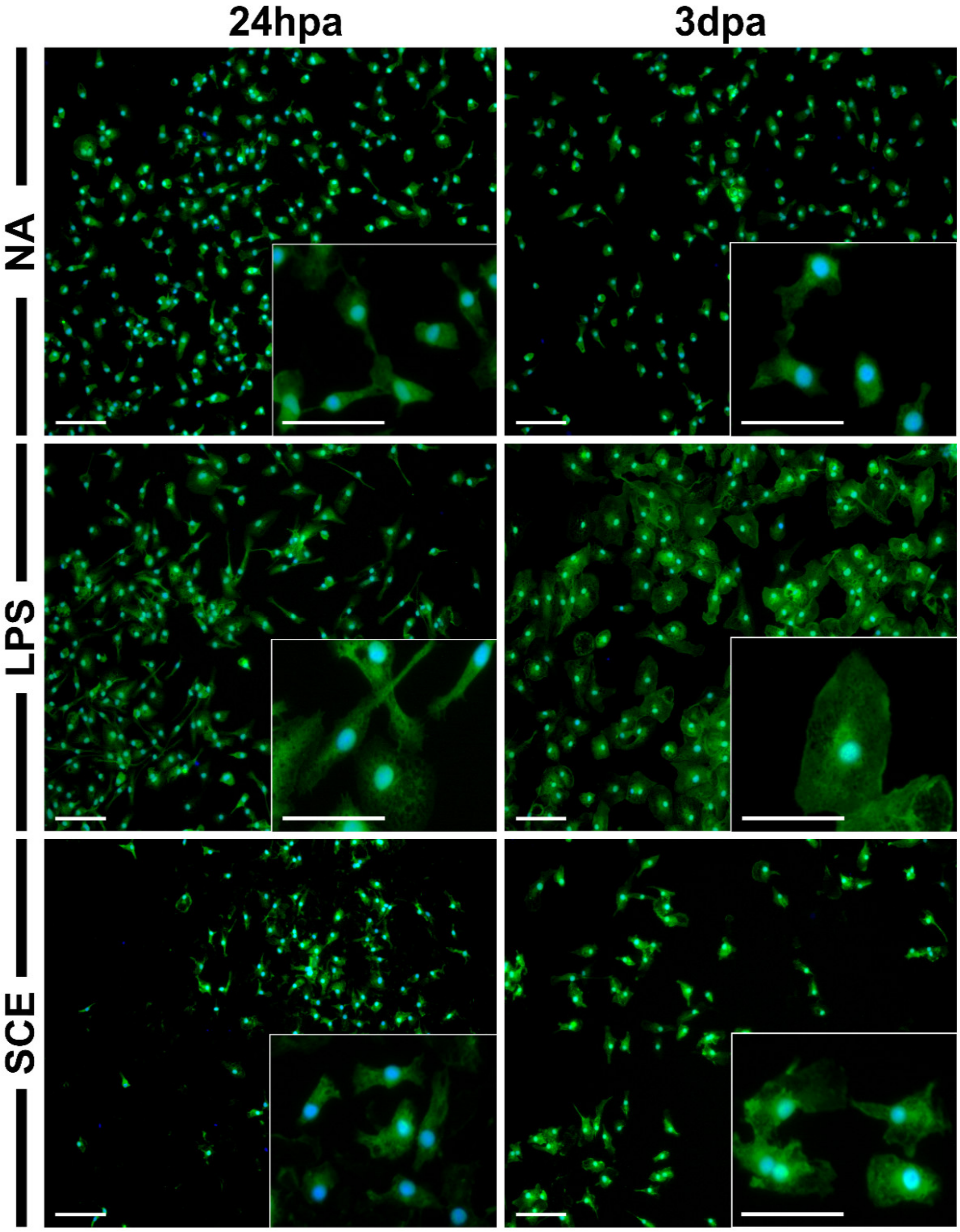

Figure S1. Microscopic analysis of microglia cell cultures. Representative images of non-activated (NA) microglial cells and after activation with 100 ng/mL of lipopolysaccharide (LPS) or with 200 µg/mL of lesioned spinal cord extract (SCE) at 24 h post-activation (hpa) and 3 days post-activation (dpa). Scale bars in low magnification images represent 100 µm, while scale bars in high magnification images represent 50 µm.

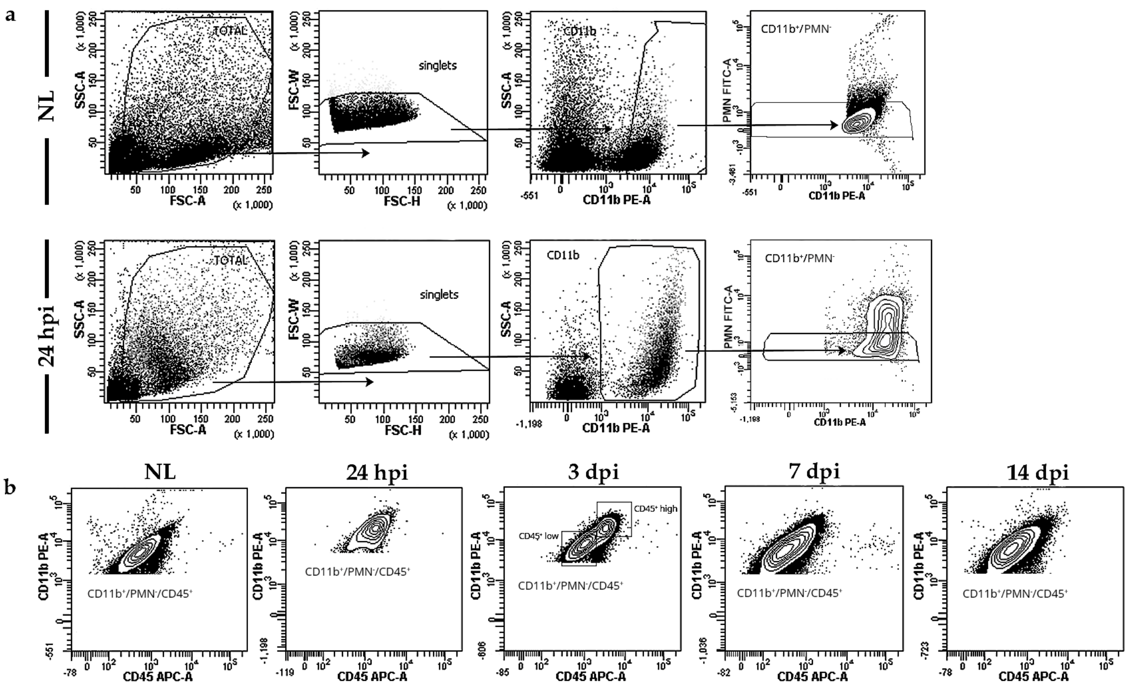

Figure S2. Gating strategy for rat microglia that were isolated by fluorescent-activated cell sorting (FACS). (**a**) Representative plots of non-lesioned (NL) and 24 h post-injured spinal cord (hpi) showing how microglia and macrophages were gated (CD11b$^+$/PMN$^-$). (**b**) Representative setting from CD45 sorting, showing how CD45$^+$ low and high subpopulations of microglia were tried to be gated. The only point of the time course where we were able to discriminate low and high CD45$^+$ subpopulations over the CD11b$^+$/PMN$^-$ cells was 3 days post-injury (dpi).
